# Mosquito-borne transmission in urban landscapes: the missing link between vector abundance and human density

**DOI:** 10.1098/rspb.2018.0826

**Published:** 2018-08-15

**Authors:** Victoria Romeo-Aznar, Richard Paul, Olivier Telle, Mercedes Pascual

**Affiliations:** 1 Department of Ecology and Evolution, University of Chicago, Chicago, IL 60637, USA; 2 Institut Pasteur, Functional Genetics of Infectious Diseases Unit, 75724 Paris Cedex 15, France; 3 Centre National de la Recherche Scientifique (CNRS), Génomique évolutive, modélisation et santé UMR 2000, 75724 Paris Cedex 15, France; 4 Centre National de la Recherche Scientifique (CNRS), Centre de Sciences Humaines (CSH), Delhi, India; 5 Center for Policy Research (CPR), Delhi, India; 6 Santa Fe Institute, Santa Fe, NM, 87501, USA

**Keywords:** vector-borne diseases, human density, dengue, mosquito spatial distribution, urban landscapes, heterogeneous environment

## Abstract

With escalating urbanization, the environmental, demographic, and socio-economic heterogeneity of urban landscapes poses a challenge to mathematical models for the transmission of vector-borne infections. Classical coupled vector–human models typically assume that mosquito abundance is either independent from, or proportional to, human population density, implying a decreasing force of infection, or *per capita* infection rate with host number. We question these assumptions by introducing an explicit dependence between host and vector densities through different recruitment functions, whose dynamical consequences we examine in a modified model formulation. Contrasting patterns in the force of infection are demonstrated, including in particular increasing trends when recruitment grows sufficiently fast with human density. Interaction of these patterns with seasonality in temperature can give rise to pronounced differences in timing, relative peak sizes, and duration of epidemics. These proposed dependencies explain empirical dengue risk patterns observed in the city of Delhi where socio-economic status has an impact on both human and mosquito densities. These observed risk trends with host density are inconsistent with current standard models. A better understanding of the connection between vector recruitment and host density is needed to address the population dynamics of mosquito-transmitted infections in urban landscapes.

## 1. Introduction

Vector-borne infections impose a major public health burden worldwide and also affect livestock and wildlife, as the result of both established and recently emergent pathogens. Globally, these pathogens account for more than 17% of all infectious diseases in humans and cause more than 700 000 deaths annually [1]. Environmental and demographic factors influencing their distribution are currently experiencing pronounced change, from modified seasons to climate trends, to expanding land use and urbanization [2,3]. Urban landscapes provide environmental settings for the persistence of urban malaria in South Asia, and the emergence, re-emergence, and establishment around the world of several infectious diseases transmitted by mosquitoes [4–7]. Most vector-borne infections are in these landscapes the product of the peridomestic distribution of the vectors [8,9]. This domiciliary coexistence results from suitable environments generated by human activities and also from the vectors' ability to adapt their life cycle to new habitats [10]. The mosquito *Aedes aegypti* provides a perfect example of this adaptation, with almost any water container serving as a potential breeding site [11]. The main vector of dengue virus, this mosquito is also responsible for the emergence of the Zika and chikungunya viruses, and for the transmission of the recently re-emerging yellow fever virus in several countries [12,13].

Rapid urbanization raises the central question of the effects of host density on these transmission systems. In particular, we can ask whether the transmission rate experienced by an individual host increases or decreases with population density [14]. Typically, in standard mathematical models for vector-borne infections formulated as extensions of the original Ross–MacDonald equations [15,16], this force of infection decreases because of the common assumptions of either a constant ratio between mosquito and human numbers (e.g. [13,17]) or their complete independence [14]. These deep-seated assumptions, either implicit or unrecognized, lack empirical justification (but see [18] for evidence in Rift Valley fever in livestock). Although they could be perhaps sufficient in temporal models for a given location, as long as comparisons are not sought across locations with differences in population density, the form of this dependence could be critical in spatio-temporal models, especially in urban landscapes. More complex dengue models that explicitly track the distribution and abundance of containers do exist [19], but these formulations require empirical specification and do not provide direct understanding of the consequences of variation in human density.

We consider here a basic modification of classical models for the population dynamics of vector-borne infection and introduce an explicit dependence between host density and vector abundance through mosquitoes’ recruitment. This modified system recognizes that increasing human densities are expected to generate increasing numbers of mosquito breeding sites and thus mosquito numbers, albeit with a likely asymptote. Within the classical framework of models, a family of functions is considered to represent a spectrum of variation in the mosquito recruitment–human density relationship and to investigate its dynamical consequences in both temporal and spatial settings. We specifically derive conditions for an increased *per capita* risk of infection with human density and demonstrate fundamental differences in transmission dynamics compared with models with standard assumptions. With empirical data on reported cases from the city of Delhi, India, evidence is presented for the existence of such a dependence across different parts of the city corresponding to different human densities and socio-economic typologies.

Consequences of variation in host numbers have been previously addressed from the perspective of density-dependent versus frequency-dependent transmission in relation to the biting behaviour of vectors [14,20]. We argue that the condition needed for density dependence and therefore for an increasing force of infection, namely a very small number of encounters between vectors and hosts (of less than one host per vector per week) [14], finds restricted application in urban landscapes. In these environments, the abundance of breeding sites rather than host encounters is likely to act as the limiting factor determining the carrying capacity of vectors. Understanding how to include this currently missing link between host and vector abundance is critical to modelling the population dynamics of vector-borne infections in the heterogeneous landscapes of today's urbanized world.

## 2. Material and methods

In our modified model, we consider that humans generate potential breeding sites in the form of water containers for the recruitment of the vector, and that this relationship can be described by a function whose specific form potentially depends on social factors. Thus, a function *V*(*N*) describing the carrying capacity of mosquitoes as a function of human density *N* is introduced in the classical formulation of coupled vector–human transmission models.

### (a) The model

As in classical epidemiological models for vector-borne infections, we consider two coupled subsystems: the first is an *SIR* (susceptible, S; infected, I; recovered, R) model for the host; the second, an *SI* (susceptible, W; infected, Z) model for the vector. The population dynamics of the system is described by the following system of equations:
2.1a
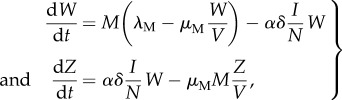

2.1b
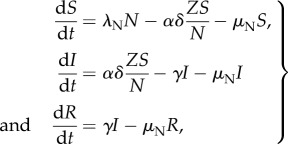
where *λ_j_* and *μ_j_* denote the birth and mortality rates (with *j = M* for vectors and *j = N* for hosts), *α* is the biting rate, *γ*, the recovery rate and *δ*, the virus transmission probability given a bite by a susceptible mosquito of an infectious human. Without loss of generality, we consider for simplicity an equal transmission probability in both directions, from host to vector and from vector to host. Although it is well known that most of these parameters depend on temperature, we initially keep them constant to begin with constant environmental conditions and then vary specific parameters seasonally as a function of temperature. (The parameter values are adapted from [21,22]. See electronic supplementary material, table S1.) From (equation (2.1*a*)), the total number of vectors, *M = W + Z*, follows a logistic equation (equation (2.2)), whose carrying capacity is proportional to the function *V*(*N*)*:*
2.2
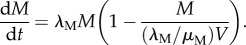


### (b) Functions *V*(*N*) and the force of infection

The specific functions *V*(*N*) considered here are motivated by the expression for the force of infection, the transmission rate per susceptible individual, which in our model is defined as FOI = *δαZ/N*. To examine different dynamical scenarios resulting from variation in host density, we seek to consider functions *V*(*N*) giving different behaviours of the force of infection with *N*.

By making a quasi-static approximation to the set of differential equations and assuming that 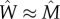 (from 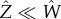), we obtain from equation (2.1)
2.3
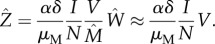
Substituting this expression for the infected vector in the FOI, we have that
2.4
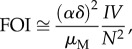
and substituting equation (2.4) into equation (2.1*b*), we obtain the following approximation for *I*:
2.5
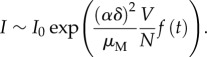
Then, from equations (2.4) and (2.5), we can write an approximate analytical expression for the force of infection itself,
2.6
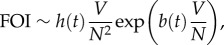
where *h*(*t*) and *b*(*t*) denote two functions of time.

Motivated by equation (2.6), we chose a family of power functions, namely 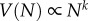. To analyse the behaviour of the FOI with *N* for different exponents *k*, we take the derivative of the approximate FOI and obtain the following equation:
2.7
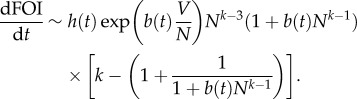
From equation (2.7), given that the factor 1 + 1/(*b*(*t*)*N*^*k*−1^ + 1) > 1 is bounded from below, we have that the FOI is a decreasing function of *N* if *k* is lower than or equal to one. This factor is also bounded from above, 2 > 1 + 1/(*b*(*t*)*N*^*k*−1^ + 1), which implies an increasing behaviour of the FOI with *N* when *k* is greater than or equal to two.

**Figure 1.**
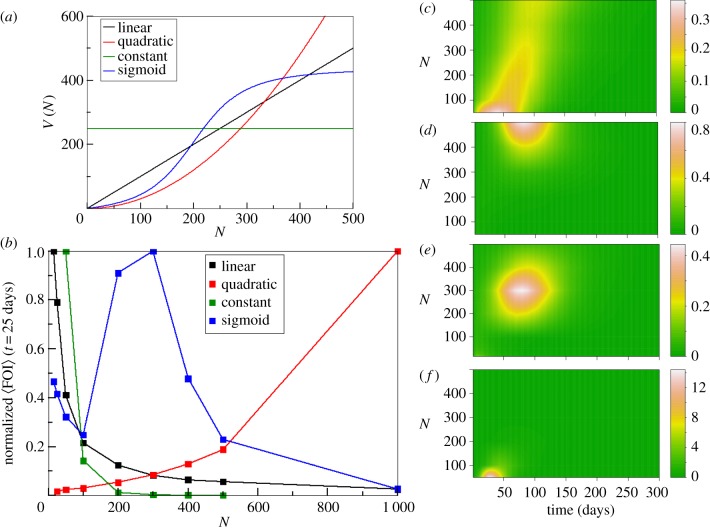
(*a*) The different functions *V*(*N*) considered in this study. (*b*) Mean value of the force of infection (over 2 000 runs for *t* = 25 days and normalized by its maximum value with respect to *N*) as a function of human density *N*, where colours represent simulations with different functions *V*(*N*). The FOI values in (*b*) correspond to a cut at *t* = 25 days of the surfaces in (*c*), (*d*), (*e*), and (*f*) for the linear, quadratic, sigmoid, and constant cases, respectively. These surface plots show the force of infection (100×) as a function of time and *N*.

Figure 1*a* illustrates the family of functions *V*(*N*) considered here (*k* = 0, 1, 2), together with an additional sigmoid curve corresponding to a type III functional form. (This additional function allows for a non-monotonic behaviour with *N*, given that we now have variation at both a higher and lower rate than quadratic in different parts of the curve.)

### (c) Temporal, seasonal, and spatial simulations

We numerically implement the full model (equation (2.1)) stochastically in continuous time, technically as a Markov (jump) process [23–25], to represent Poisson processes with exponentially distributed times (for faster simulation, we use the approximation method described in [26]). A stochastic implementation allows us to take into account the effect of extinction outcomes on the means presented below and to specifically consider probabilistic quantities when analysing the Delhi data. To introduce seasonality, we generated periodic vector abundance by sinusoidally forcing the mortality and birth rates of the mosquitoes, mimicking the effect of seasonal temperature variation.

For the spatial simulations, a grid of units represents two-dimensional space, and within each unit, both vector and host populations follow the above transmission model in system (2.1). The coupling between units is implemented via mosquito movement to neighbouring units, with the flight rate depending on the local carrying capacity [21] (see electronic supplementary material, table S1 for details).

### (d) Empirical data and analysis

To explore empirical evidence for the hypothetical dependence represented by *V*(*N*), we analysed yearly reported dengue cases for the city of Delhi (India) for years 2008, 2009, and 2010 [27]. This yearly dengue data are published and described in detail in [27] as part of a Geographical Information System for the city constructed at a spatial scale of 250 m × 250 m (the spatial ‘unit’). In this previous study, each of these 10 676 units was assigned to one of eight socio-economic classes based on a principal component analysis (PCA) classification. The socio-economic and demographic characteristics for the PCA analysis consisted of six variables, four of which were selected from the property tax information for Delhi's colonies as known risk factors associated with other infectious diseases. These variables are level of infrastructure, economic status of resident, type of colony, and total property tax score. Two additional variables consist of the percentage of land dedicated to industry and the population density estimated from the 2011 census and remote sensing. The spatial resolution was chosen to create homogeneous units as described in [27]. The resulting groupings of these units are called ‘typologies’ hereafter and include Rich, Deprived Low Density (or Dep. LD), Deprived Medium Density (or Dep. MD), Deprived High Density (Dep. HD) as well as Planned, Industrial, Cantonment, and Rural Periphery [27]. The Deprived typologies as their name indicates correspond to conditions of low wealth. We focus here on typologies with a sufficient number of units for the analysis described below; these include in particular Rich and Deprived LD and MD.

Because of the noisy nature of the data with high under-reporting, frequent asymptomatic infections [28], and only a few reported cases at the level of units, as well as common extinctions and absence of cases, we assessed the local capacity of units within each typology to propagate infection. Given that the arrival of an infected individual is necessary to generate infected hosts inside a unit, we considered that all the units with at least one reported case in a given year have received an external infection, and that units with more than one case are likely to have experienced local transmission. In other words, we consider that, in the span of one year, there is a very low probability that more than one person has contracted the infection outside their unit. This assumption is particularly sensible for 2008 and 2009, years with a low virus circulation in the city compared with 2010. Then, for a given typology *Ty*, a unit *u* with a population density *N* has a probability *p* of generating infected people locally, given by
2.8
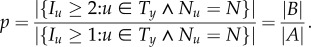
For each typology, the units where infections are present are grouped by human density *N* (set *A*), and from this set, the units with two or more infections are selected (set *B*). Then, *p* is computed as the ratio between the number of elements of these two sets (equation (2.8); for a detailed description, see electronic supplementary material, figure S1). Based on our finding below that *p* depends on the ratio of vectors to humans and is invariant with the function *V*(*N*), we are able to estimate the power function *V*(*N*) that best fits the empirical variation of *p* with *N*. This allows us to interrogate the data on whether *V*(*N*) increases faster than linearly, with consequences for an increased FOI with *N*.

## 3. Results

### (a) The force of infection

The respective FOI from the simulations with the different *V*(*N*) curves (figure 1*a*) are plotted in figure 1*b* as a function of *N* and for a given time chosen to fall before the peak of the epidemic. (As the FOI varies dynamically, these curves represent a time section through the corresponding surface representing the variation of this quantity with *N* and time; figure 1*c–f*.) We obtain different trends with *N* and, therefore, different dynamic behaviours for different population densities. For the linear and constant cases, the FOI decreases when *N* grows, implying a lower rate of infection per susceptible host when *N* is large. The opposite behaviour is obtained when *V*(*N*) is quadratic and for the sigmoid case, the FOI exhibits a non-monotonic behaviour with *N*. In particular, the FOI does not necessarily decrease as expected from existing models. The described trends of the FOI with *N* are robust to variation in other model parameters (see electronic supplementary material, figure S2 as an example of variation in the biting rate).

Vector recruitment also affects other important epidemiological quantities that do not vary dynamically under fixed external conditions, in particular the basic reproductive number *R*_0_. (We refer here strictly to *R*_0_ when the population as a whole is susceptible.) From the system equations (equation (2.1)), we have 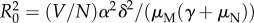 which, as known, shows that *R*_0_ depends on the vector–host ratio. As a result, variation of *R*_0_ with *N* is not the same as that of the FOI. For example, *R*_0_ remains constant with *N*, whereas the FOI decreases, for linear *V*(*N*) (see electronic supplementary material, figure S3).

### (b) Temporal and seasonal dynamics

**Figure 2.**
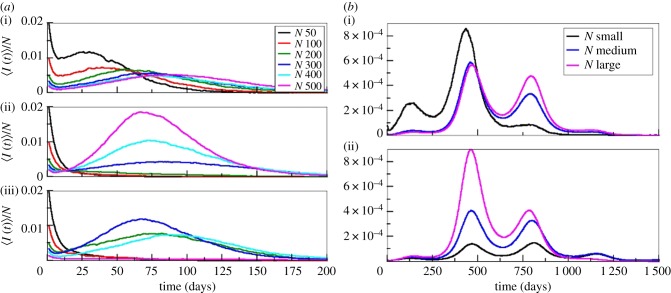
Temporal evolution of incidence mean value for the non-spatial model. (*a*) Without seasonality (constant temperature) for the linear (i), quadratic (ii), and sigmoid (iii) function *V*(*N*). (*b*) With seasonality (temperature forcing, annual pattern), for linear (i) and quadratic (ii) functions *V*(*N*) (see electronic supplementary material, figure S4 for plots with 90% confidence level).

Because the FOI is closely associated with system dynamics, the above-described variation with *N* should result in distinct patterns of the temporal evolution of infection. In figure 2*a*, the different behaviours of incidence dynamics with *N* for the linear, quadratic, and sigmoid cases are shown. For the linear case, because the force of infection decreases with *N*, the time to the epidemic peak increases with *N*. By contrast, for the quadratic, case, the initial intensity of the epidemic decreases with *N*, with a peak delay of 10 days for *N* = 400 and 25 days for *N* = 300 relative to *N* = 500. For sigmoid *V*(*N*), outbreaks only occur for medium *N* values and are absent for smaller and larger *N*. Consistent with its FOI peak, medium-sized populations (*N* = 300) have a larger initial intensity of infection (and earliest peak timing).

The above dynamics are largely ruled by the depletion of susceptible hosts. Another important mechanism behind the turnaround of epidemics in vector-borne diseases is the strong dependence of the mosquito life cycle and viral development within the mosquito on temperature [29,30]. The effect of FOI on initial epidemic intensity can give rise to more complex dynamical patterns when environmental conditions vary over time. With seasonal forcing, resulting average values of incidence show patterns of repeated outbreaks, characteristic of vector-borne infections, with the relative size of the annual peaks varying with *N* in a way that depends closely on *V*(*N*) (figure 2*b*). For example, in the linear case, although the largest first peak is always obtained for small *N*, the size of the subsequent peaks depends on *N* and increases for medium and large *N*. A completely different dynamical behaviour is obtained for the quadratic case, where peak size increases with *N*. In contrast to the linear case, for quadratic *V*(*N*), the importance of the initial peak inverses with *N*, and the first and subsequent peaks can become comparable, especially for small *N*. A relative later temporal peak is associated with smaller values of *N*.

### (c) Spatio-temporal dynamics

A spatial framework allows us to consider the effects of population density distribution through the vector's carrying capacity, while keeping the total population size constant. In particular, we explore emerging patterns resulting from a given population distribution for different functions of *V*(*N*). Results illustrate the potential importance of the *V*(*N*) dependence with one particular and common pattern of population distribution and a simple assumption about spatial coupling.

For the spatial framework a population density decreasing from the grid centre, and linear, constant, and quadratic functions *V*(*N*) were considered in the simulations. All initial conditions consist of susceptible populations and a single infected host in the centre of the grid. For the non-seasonal case (figure 4*a*(i)), the epidemic peak timing (*T*), as well as the beginning and the end of the outbreak vary significantly with *V*(*N*). Here, two spatial effects from the centre to the periphery of the grid are at play, which act together to give the aggregated temporal trajectory, that of FOI and *V*/*N*. An early and sharp epidemic (at *T* = 170 days) is obtained for the quadratic case whose initial FOI is greater than that for the linear and constant cases, given the host distribution. The constant *V*(*N*) exhibits a later peak (*T* = 435 days) than the linear case (*T* = 350 days) because, in addition to the FOI effect, disease spread occurs under a decreasing vector–human ratio, whereas this ratio remains constant for linear *V*(*N*). As a consequence, for constant *V*(*N*), the access to units that can produce a large fraction of infected hosts takes time, inasmuch the epidemic spreads by neighbouring coupling only.

**Figure 3.**
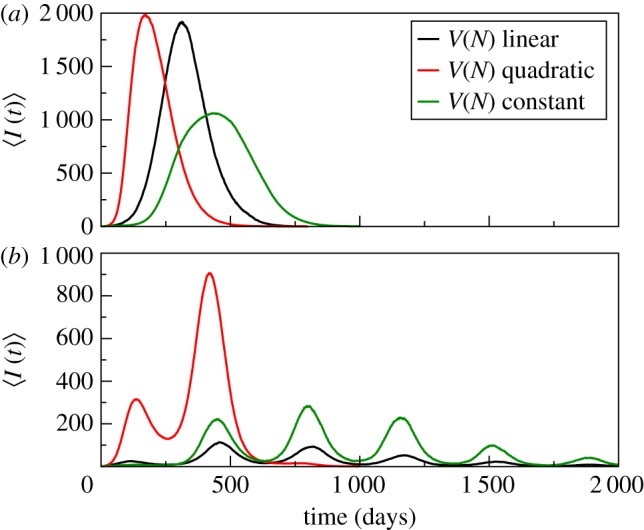
Temporal evolution of the total number of infected humans in the grid (mean value), without (*a*) and with (*b*) seasonal temperature forcing (see electronic supplementary material, figure S5 for the same plots with the 90% confidence level).

As a consequence of these differential dynamics, we obtain very distinct temporal patterns when seasonal forcing by temperature is applied (figure 3*b*). For example, for a quadratic function *V*(*N*), the early and sharp epidemic under constant temperature generates two early, large outbreaks. By contrast, a later and broader/flatter curve translates into a large number of smaller outbreaks as generated by linear and constant functions of *V*/*N*. Given that the total numbers of both humans and vectors are the same across the simulations, these emergent patterns result from the combination of variation in the local force of infection with the spatial spread of the infection.

### (d) Empirical patterns and estimated *V*(*N*) from Delhi data

**Figure 4.**
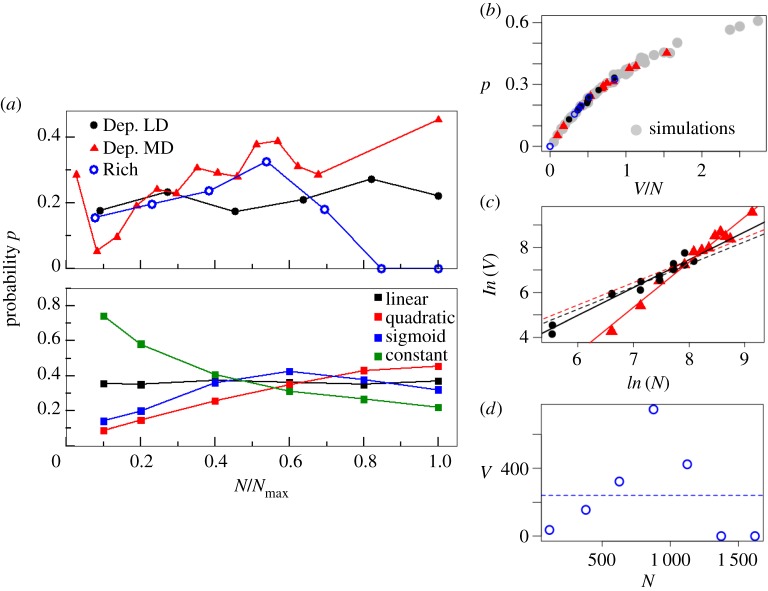
(*a*) Probability *p* that a unit generates local infections as a function of population density (normalized by its maximum value to compare the curves on the same scale; see electronic supplementary material, figure S6 for *p* as a function of *N* and for separate years). (i) Epidemic dengue data from Delhi, where *N*_max_ is the maximum human density (humans per unit) of the corresponding typology. (ii) Simulations where *N*_max_ correspond to the maximum value of the studied *N*. (*b*) *p* is plotted as a function of *V/N*, for both the simulations (in grey, regardless of function) and the data (in the colours used throughout the figures for the different functions). From the empirical values of *p* and this curve, the corresponding empirical values of *V*/*N* can be computed. (*c*) The natural logarithm of *V* is plotted as a function of the natural logarithm of *N*. The dots correspond to data values, with red triangles for Dep. MD and black circles for Dep. LD and Planned typologies. (The data from the planned units were added to those from Dep. LD because their observed *p* values and behaviour with *N* are similar; see electronic supplementary material, figure S6A. This allows us to increase the number of points for the estimation of *k.*) The solid lines correspond to the best linear fit with least-square regression, and the dashed lines, to the best fit with a fixed slope of one. For the Dep. MD analyses, we assumed that its first point (for the smallest *N*) is an outlier. If we do not remove it, however, a value of *k* = 1.45 ± 0.17 (plot and linear fit are shown in electronic supplementary material, figure S7) is obtained. (*d*) Values of *V* obtained from the observed *p* are plotted as a function of *N* for the Rich typology. The dashed line corresponds to the expected values under the assumption of a constant ratio between *V* and *N*.

Figure 4*a*(i) shows the probability *p* (see equation (2.8)) plotted as a function of *N*/*N*_max_ for the Dep. MD, Dep. LD, and Rich typologies for the Delhi data. Depending on the typology, within its characteristic population density range, we observe different trends for the probability *p*. For the Dep. MD typology, *p* increases with *N*; for Dep. LD, this quantity remains approximately constant, and for the Rich typology, it increases and then decreases.

We then asked whether our temporal model is capable of capturing these different patterns. We considered that each unit experiences well-mixed conditions and, because *p* is the probability that a unit generates infections locally upon arrival of an external disease ‘spark’, we modelled units independently from each other with a single infected human as the initial condition. For each *V*(*N*), figure 4*a*(ii) shows the probability of a unit exhibiting more than one infected host as a function of *N*/*N*_max_. These simulated patterns qualitatively capture the empirical trends, showing an increasing, a constant, and non-monotonic (up and down) behaviour, for the quadratic, constant, and sigmoid functions, respectively. In particular, we highlight that the typical assumption of the number of breeding sites, and hence mosquito population size, being independent from host density, would generate a decreasing behaviour of *p* with *N*/*N*_max_*.* Figure 4*a* suggests that this pattern does not hold for any of the trends obtained from the empirical data.

To estimate the power-function dependence of *V* with *N* (*V*(*N*) = *cN^k^*) that best explains these data for a given typology, we first estimated the empirical mean value of *V* for each set of spatial units with similar *N*. In this way, we can obtain a set of empirical points (*V*, *N*) to fit the corresponding power function. To this end, we first observe that as expected from [31], the probability *p* from the simulations depends only on *V*/*N* and not on the specific function *V*(*N*) (figure 3*b*). Thus, we can use this curve to compute from the observed value of *p* (coloured points in figure 4*b*) the corresponding empirical value of *V*/*N.* We then use this ratio itself to compute *V* by simple multiplication by the known value of *N*. The exponent *k* can finally be estimated by a linear regression of ln(*V*) as a function of ln(*N*) (figure 4*c*). We obtain values *k* = 2.01 ± 0.10 for the Dep. MD case, and *k* = 1.24 *±* 0.08 for the Dep. LD, to which we have added the data from the planned typology as explained in figure 4*c*. Given the apparent non-monotonic behaviour of *V* with *N* for the Rich typology (figure 4*d*), we would need to consider a piecewise linear dependence. Because this would result, however, into too few points in each section, especially for the decreasing branch, we did not fit *V*(*N*) for this case.

## 4. Discussion

It is well known that the force of infection is a key quantity for epidemiological dynamics. Thus, its relation with human density should be critical to epidemic spread, particularly in urban landscapes and other settings with heterogeneous population distributions. With a simple modification of the classical equations for vector-borne infections, we showed that the effect of human density on the force of infection depends strongly on the dependence of mosquitoes and human numbers represented by a function *V*(*N*)*.* In particular, a dense environment can be misrepresented by a dilution of the infection probability rate as expected from the common assumption of independent or linear human–vector recruitment. Even under a constant temperature environment, we showed that *V*(*N*) determines epidemic peak timing, duration, and beginning of the outbreak. Although a non-seasonal environment is clearly unrealistic, these results translated into very different incidence patterns under seasonal temperature forcing. These kinds of patterns in interannual variation are typically addressed in terms of climate variability, for example in temperature [32], and in some cases also as the result of susceptible depletion. We show, however, that different dependencies of the human–mosquito density relationship introduce significant variation in the timing and size of consecutive outbreaks, whether in a purely temporal or in a spatio-temporal context.

Because the function *V*(*N*) results from a variety of socio-economic characteristics mediating the effect of density on vector abundance, its shape would be expected to vary within a city and especially in developing countries. We have provided evidence for density-dependent risk patterns within Delhi, where the probability that a spatial unit in the city develops infections locally depends both on its population density and typology. This variation in dengue risk pattern *V*(*N*) suggests different dependencies of vector abundance on human density for the different socio-economic typologies, with significant disease consequences. The estimated quadratic dependence of the part of the city that was previously classified as Deprived MD clearly departs from the common assumptions made to formulate models of vector-borne transmission. Poor socio-economic conditions in Delhi are associated with a lack of access to regular pipe water and solid waste [33]. As a result, the storage of water and the proliferation of potential containers for rainfall would produce breeding sites for mosquitoes and therefore higher abundances with more humans. For the Deprived LD case, the overall lower population density range could explain the estimated lower exponent, whose value would still imply a faster than linear production of breeding sites. For the Rich typology, our results suggest that a non-monotonic function *V*(*N*) may underlie the risk patterns, implying a saturation effect when population density is large enough. Under better socio-economic conditions and better access to water and urban services, an increasing population density may not keep increasing the availability of breeding sites. The numbers of water containers, such as flower pots, air conditioners, and gutters, do not keep increasing after a given number of humans is reached. With an increasing presence of tall buildings, water containers could be also less likely to produce mosquitoes [34,35]. Interestingly, a sigmoid relationship between humans and vectors has been proposed in [36] for an analysis of dengue fever in Vietnam.

Our estimation of *V*(*N*) for different parts of the city relied on the observation that the probability *p* of local transmission is invariant with the specific shape of this function, and only depends on the ratio of *V* and *N*. This pattern is consistent with the expression derived by Bartlett [31] for the probability of a major outbreak, which is in turn related to the known relationship of *R*_0_ with this ratio [37] (see electronic supplementary material, figure S3).

Different trends for *R*_0_ with *N* have already been debated in terms of frequency versus density-dependent contacts between host and vectors [38,39]. Because contacts are considered the result of vector biting behaviour, these conflicting predictions are reconciled by arguing that different forms of the transmission term apply biologically only at certain population densities [20]. The empirical patterns obtained for Delhi would imply biting rate dependencies other than those that have been proposed, and would be difficult to justify solely on the basis of these arguments.

The shape of the relationship between the force of infection and human density has been discussed for the different assumptions of frequency-dependent and density-dependent biting rates, as well as some intermediate cases, resulting from behavioural considerations on the vector [14]. Although a density-dependent assumption can provide an increasing FOI with *N*, the conditions for this pattern to hold appear restricted to an unrealistic number of humans per mosquito. For the general transmission rate derived by the authors in [14] from behavioural considerations, a density-dependent scenario applies when 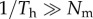, where *T*_h_ is the handling time (related to the gonotrophic cycle, [29,40]) and *N*_m_ is the number of humans encountered by mosquitoes per unit time. By assuming that *T*_h_ ∼ 1 day, we obtain 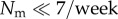 (≪ indicating less than one order of magnitude) and, therefore, in a span of a week vectors should not be able to find more than a single host for the hypothesis of a density-dependent biting rate behaviour to apply.

The spatial distribution of mosquitoes' breeding sites is key to understanding the spread of vector-borne infections as it introduces an important source of spatial heterogeneity [41]. For such heterogeneous environments, models based on mosquito blood-meal search have been proposed [40,42]. For example, the authors in [42] studied configurations resulting from mosquitoes emerging at one location and spreading subsequently driven by blood ingestion. This approach would not apply at city scales for many vectors because they would have to be able to fly long distances or be very long-lived. The *A. aegypti* mosquito does not fly more than a hundred metres from its breeding site under normal conditions [43,44]. The authors of [42] recognize that allowing the mosquito to oviposit everywhere is a critical assumption of their model, needed to understand where and when adult mosquitoes will emerge.

The growth of urban populations and climate change define major environmental challenges of this century. In particular, urban environments introduce highly heterogeneous environmental and socio-economic conditions that can affect the population dynamics of vector-borne infections. Although high-resolution data on human distributions and their socio-economic conditions are becoming increasingly accessible, spatial information is clearly much more limited and challenging to obtain for vectors. The proposed function relating vector and human numbers therefore provides one empirical approach to represent important spatial heterogeneities in mathematical models for the population dynamics of vector-borne infections in cities. Such functions can be directly examined from field measurements of vector abundance and recruitment, or indirectly derived from disease surveillance data and associated joint distributions of human densities and socio-economic conditions. Their effects on spatio-temporal dynamics should be further investigated in models with different spatial structures and more realistic spatial landscapes representative of specific urban environments. Although for simplicity, we have considered only neighbouring coupling, there is evidence on the importance of human movement for the transmission of dengue, and the exploration of more realistic connectivity networks is becoming increasingly possible [45,46]. We expect our result of pronounced differences in temporal patterns of epidemic spread to still hold across different functions *V*(*N*).

As illustrated here with the seasonally forced simulations, this dependence can have profound impact on the interplay of climate forcing and disease dynamics in urban environments. The importance of including urban characteristics to better understand how increasing temperatures would affect infectious diseases has already been argued by others [47–49]. We underscore that such an agenda should include population density as a central variable, with the possibility that social and environmental characteristics can completely reverse the expected pattern of decreased risk per individual as human density increases.

## Supplementary Material

Supplementary Figures and Parameter Table

## Data accessibility

The data used in this work are publicly available through the publication by Telle *et al*. ([27], electronic supplementary material). The C code to simulate the model is available via GitHub at https://github.com/pascualgroup/VBM.git.

## References

[1] World Health Organization. *Vector-borne diseases fact sheet* 2017 See http://www.who.int/mediacentre/factsheets/fs387/en/ (last accessed 17 January 2018).

[2] Kittayapong P, Yoksan S, Chansang U, Chansang C, Bhumiratana A 2008 Suppression of dengue transmission by application of integrated vector control strategies at sero-positive GIS-based foci. Am. J. Trop. Med. Hyg. 78, 70–76. (10.4269/ajtmh.2008.78.70) 18187787

[3] Gubler DJ 2011 Dengue, urbanization and globalization: the unholy trinity of the 21st century. Trop. Med. Health 39(4 Suppl), S3–11. (10.2149/tmh.2011-S05) PMC3317603 22500131

[4] Gubler DJ 2002 Epidemic dengue/dengue hemorrhagic fever as a public health, social and economic problem in the 21st century. Trends Microbiol. 10, 100–103. (10.1016/S0966-842X(01)02288-0) 11827812

[5] Kraemer MU, Perkins TA, Cummings DA, Zakar R, Hay SI, Smith DL, Reiner RC 2015 Big city, small world: density, contact rates, and transmission of dengue across Pakistan. J. Royal Soc. Interface 12, 20150468 (10.1098/rsif.2015.0468) PMC4614486 26468065

[6] Hennessey M, Fischer M, Staples JE 2016 Zika virus spreads to new areas—region of the Americas, May 2015–January 2016. Am. J. Transplant. 16, 1031–1034. (10.1111/ajt.13743) 26820163

[7] Fischer M, Staples JE 2014 Chikungunya virus spreads in the Americas—Caribbean and South America, 2013–2014. MMWR 63, 500. 24898168 PMC5779358

[8] Morrison AC, Zielinski-Gutierrez E, Scott TW, Rosenberg R 2008 Defining challenges and proposing solutions for control of the virus vector *Aedes aegypti*. PLoS Med. 5, e68 (10.1371/journal.pmed.0050068) 18351798 PMC2267811

[9] El-Badry AA, Al Ali KH 2010 Prevalence and seasonal distribution of dengue mosquito, *Aedes aegypti* (Diptera: Culicidae) in Al-Madinah Al-Munawwarah, Saudi Arabia. J. Ethol. 7, 80–88. (10.3923/je.2010.80.88)

[10] Weaver SC, Reisen WK 2010 Present and future arboviral threats. Antiviral Res. 85, 328–345. (10.1016/j.antiviral.2009.10.008) 19857523 PMC2815176

[11] Pramanik MK, Aditya G, Raut SK 2007 Seasonal prevalence of *Aedes aegypti* immatures in Kolkata, India. Southeast Asian J. Trop. Med. Public Health 38, 442. 17877217

[12] Kraemer MU et al. 2015 The global distribution of the arbovirus vectors *Aedes aegypti* and *Ae. albopictus*. eLife 4, e08347 (10.7554/eLife.08347) 26126267 PMC4493616

[13] Caminade C, Turner J, Metelmann S, Hesson JC, Blagrove MSC, Solomon T, Morse AP, Baylis M 2017 Global risk model for vector-borne transmission of Zika virus reveals the role of El Niño 2015. Proc. Natl Acad. Sci. USA 114, 119–124. (10.1073/pnas.1614303114) 27994145 PMC5224381

[14] Antonovics J, Iwasa Y, Hassell MP 1995 A generalized model of parasitoid, venereal, and vector-based transmission processes. Am. Naturalist. 145, 661–675. (10.1086/285761)

[15] Macdonald G 1957 The epidemiology and control of malaria. Oxford, UK: Oxford University Press.

[16] Reiner RC et al. 2013 A systematic review of mathematical models of mosquito-borne pathogen transmission: 1970–2010. J. Royal Soc. Interface 10, 20120921 (10.1098/rsif.2012.0921) PMC3627099 23407571

[17] Dye C, Wolpert DM 1988 Earthquakes, influenza and cycles of Indian kala-azar. Trans. R Soc. Trop. Med. Hyg. 82, 843–850. (10.1016/0035-9203(88)90013-2) 3256984

[18] Fischer EA, Boender GJ, Nodelijk G, De Koeijer AA, Van Roermund HJ 2013 The transmission potential of Rift Valley fever virus among livestock in the Netherlands: a modelling study. BMC Vet. Res. 44, 58 (10.1186/1297-9716-44-58) PMC3733972 23876054

[19] Magori K, Legros M, Puente ME, Focks DA, Scott TW, Lloyd AL, Gould F 2009 Skeeter buster: a stochastic, spatially explicit modeling tool for studying *Aedes aegypti* population replacement and population suppression strategies. PLoS Negl. Trop. Dis. 3, e508 (10.1371/journal.pntd.0000508) 19721700 PMC2728493

[20] Wonham MJ, Lewis MA, Renclawowicz J, van den Driessche P 2006 Transmission assumptions generate conflicting predictions in host–vector disease models: a case study in West Nile virus. Ecol. Lett. 9, 706–725. (10.1111/j.1461-0248.2006.00912.x) 16706915

[21] Otero M, Solari HG 2010 Stochastic eco-epidemiological model of dengue disease transmission by *Aedes aegypti* mosquito. Math. Biosci. 223, 32–46. (10.1016/j.mbs.2009.10.005) 19861133

[22] Romeo Aznar V, Otero M, De Majo MS, Fischer S, Solari HG 2013 Modeling the complex hatching and development of *Aedes aegypti* in temperate climates. Ecol. Model. 253, 44–55. (10.1016/j.ecolmodel.2012.12.004)

[23] Kolmogoroff A 1931 Über die analytischen Methoden in der Wahrscheinlichkeitsrechnung. Math. Ann. 104, 415–458. (10.1007/BF01457949)

[24] Feller W 1940 On the integro-differential equations of purely discontinuous Markoff processes. Transactions of the American Mathematical Society. 48(3), 488–515. (10.2307/1990095)

[25] Durrett R, Durrett R 2016 Essentials of stochastic processes. Berlin, Germany: Springer.

[26] Solari HG, Natiello MA 2003 Stochastic population dynamics: the Poisson approximation. Phys. Rev. E 67, 031918 (10.1103/PhysRevE.67.031918) 12689112

[27] Telle O, Vaguet A, Yadav NK, Lefebvre B, Daudé E, Paul RE, Cebeillac A, Nagpal BN. 2016 The spread of dengue in an endemic urban milieu—the case of Delhi, India PLoS ONE 11, e0146539 (10.1371/journal.pone.0146539) 26808518 PMC4726601

[28] Vikram K et al. 2016 An epidemiological study of Dengue in Delhi, India. Acta Trop. 153, 21–27. (10.1016/j.actatropica.2015.09.025) 26433076

[29] Christophers S 1960 Aëdes aegypti (L.) the yellow fever mosquito: its life history, bionomics and structure. London, UK: Cambridge University Press.

[30] Tun-Lin W, Burkot TR, Kay BH 2000 Effects of temperature and larval diet on development rates and survival of the dengue vector *Aedes aegypti* in north Queensland, Australia. Med. Vet. Entomol. 14, 31–37. (10.1046/j.1365-2915.2000.00207.x) 10759309

[31] Bartlett MS 1964 The relevance of stochastic models for large-scale epidemiological phenomena. J. R. Stat. Soc. Ser. C Appl. Stat. 13, 2–8. (10.2307/2985217)

[32] Cuong HQ et al. 2013 Spatiotemporal dynamics of dengue epidemics, southern Vietnam. Emerg. Infect. Dis. 19, 945 (10.3201/eid1906.121323) 23735713 PMC3713821

[33] Vikram K et al. 2015 Comparison of *Ae. aegypti* breeding in localities of different socio-economic groups of Delhi, India. Int. J. Mosq. Res. 2, 83–88.

[34] Goh KT 1998 Dengue in Singapore, pp. 213–242. Singapore: Institute of Environmental Epidemiology, Ministry of the Environment.

[35] Carbajo AE, Curto SI, Schweigmann NJ 2006 Spatial distribution pattern of oviposition in the mosquito *Aedes aegypti* in relation to urbanization in Buenos Aires: southern fringe bionomics of an introduced vector. Med. Vet. Entomol. 20, 209–218. (10.1111/j.1365-2915.2006.00625.x) 16871702

[36] Schmidt W-P et al. 2011 Population density, water supply, and the risk of dengue fever in Vietnam: cohort study and spatial analysis. PLoS Med. 8, e1001082 (10.1371/journal.pmed.1001082) 21918642 PMC3168879

[37] Lloyd AL, Zhang J, Root AM 2007 Stochasticity and heterogeneity in host–vector models. J. R. Soc. Interface 4, 851–863. (10.1098/rsif.2007.1064) 17580290 PMC2394551

[38] Wood SN, Thomas MB 1999 Super-sensitivity to structure in biological models. Proc. R. Soc. Lond. B 266, 565–570. (10.1098/rspb.1999.0673)

[39] McCallum H, Barlow N, Hone J 2001 How should pathogen transmission be modelled? Trends Ecol. Evol. 16, 295–300. (10.1016/S0169-5347(01)02144-9) 11369107

[40] Sota T, Mogi M 1989 Effectiveness of zooprophylaxis in malaria control: a theoretical inquiry, with a model for mosquito populations with two bloodmeal hosts. Med. Vet. Entomol. 3, 337–345. (10.1111/j.1365-2915.1989.tb00240.x) 2519684

[41] Reisen WK 2010 Landscape epidemiology of vector-borne diseases. Annu. Rev. Entomol. 55, 461–483. (10.1146/annurev-ento-112408-085419) 19737082

[42] Smith DL, Dushoff J, McKenzie FE 2004 The risk of a mosquito-borne infection in a heterogeneous environment. PLoS Biol. 2, e368 (10.1371/journal.pbio.0020368) 15510228 PMC524252

[43] Salje H et al. 2012 Revealing the microscale spatial signature of dengue transmission and immunity in an urban population. Proc. Natl Acad. Sci. USA 109, 9535–9538. (10.1073/pnas.1120621109) 22645364 PMC3386131

[44] Bergero PE, Ruggerio CA, Lombardo R, Schweigmann NJ, Solari HG 2013 Dispersal of *Aedes aegypti*: field study in temperate areas using a novel method. J. Vector Borne Dis. 50, 163. 24220074

[45] Wesolowski A, Qureshi T, Boni MF, Sundsøy PR, Johansson MA, Rasheed SB, Engø-Monsen K, Buckee CO 2015 Impact of human mobility on the emergence of dengue epidemics in Pakistan. Proc. Natl Acad. Sci. USA 112, 11 887–11 892. (10.1073/pnas.1504964112) PMC4586847 26351662

[46] Stoddard ST et al. 2013 House-to-house human movement drives dengue virus transmission. Proc. Natl Acad. Sci. USA 110, 994–999. (10.1073/pnas.1213349110) 23277539 PMC3549073

[47] Reiter P 2001 Climate change and mosquito-borne disease. Environ. Health Perspect. 109(Suppl 1), 141 (10.1289/ehp.01109s1141) 11250812 PMC1240549

[48] Medlock JM, Leach SA 2015 Effect of climate change on vector-borne disease risk in the UK. Lancet Infect. Dis. 15, 721–730. (10.1016/S1473-3099(15)70091-5) 25808458

[49] Ogden NH, Lindsay LR 2016 Effects of climate and climate change on vectors and vector-borne diseases: ticks are different. Trends Parasitol. 32, 646–656. (10.1016/j.pt.2016.04.015) 27260548

