# Supplementary material for: Mosquito-borne transmission in urban landscapes: the missing link between vector abundance and human density
Source: Proc Biol Sci. 2018 Aug 15;285(1884):20180826. doi: 10.1098/rspb.2018.0826 (PMC6111166; doi:10.1098/rspb.2018.0826)
Supplement: Supplementary Figures and Parameter Table [file rspb20180826supp1.pdf]

Electronic supplementary material

Mosquito-borne transmission in urban landscapes: the missing link between vector abundance and human density

Victoria Romeo-Aznar[1], Richard Paul[2,3], Olivier Telle[4] and Mercedes Pascual[1]

[1]Department of Ecology and Evolution, University of Chicago, Chicago IL, USA 60637.

[2]Institut Pasteur, Functional Genetics of Infectious Diseases Unit, 75724 Paris Cedex 15, France.

[3]Centre National de la Recherche Scientifique (CNRS), Génomique évolutive, modélisation et santé UMR 2000, 75724 Paris Cedex 15, France.

[4]Centre National de la Recherche Scientifique (CNRS), CSH, Delhi, Inde.

doi: 10.1098/rspb.2018.0826

Parameter	Value	Description
λ_N	1/(70*365) days^{-1}	Humans' birth rate
μ_N	1/(70*365) days^{-1}	Humans' mortality rate
γ	1/5 days^{-1}	Recovery rate
δ	0.75	virus transmission probability given a mosquito bite
α	0.23 days^{-1}	Biting rate
Without temperature		
λ_M	0.09 days^{-1}	Mosquitoes' birth rate
μ_M	0.09 days^{-1}	Mosquitoes' mortality rate
With temperature (T)		
$\lambda_M(T)$	0.003 T days^{-1} C^{o-1}	Mosquitoes' birth rate
$\mu_M(T)$	(0.08326+e$^{-T/6\,^{o}C}$) days^{-1}	Mosquitoes' mortality rate
Spatial		

F(V(N))	$(0.059+0.342822071\ e^{-0.003777299\ V(N)})$ days^{-1}	Mosquitoes' spatial-dispersion rate
Table S1. Model parameters values (see main text). Parameter values are the same for all models. The mortality and birth rates of mosquitoes are the two parameters that vary periodically with temperature. In the spatial model, *F* is the emigration rate from a spatial unit per mosquito. The values are adapted from Otero & Solari, 2010 and Romeo Aznar et al., 2013.		

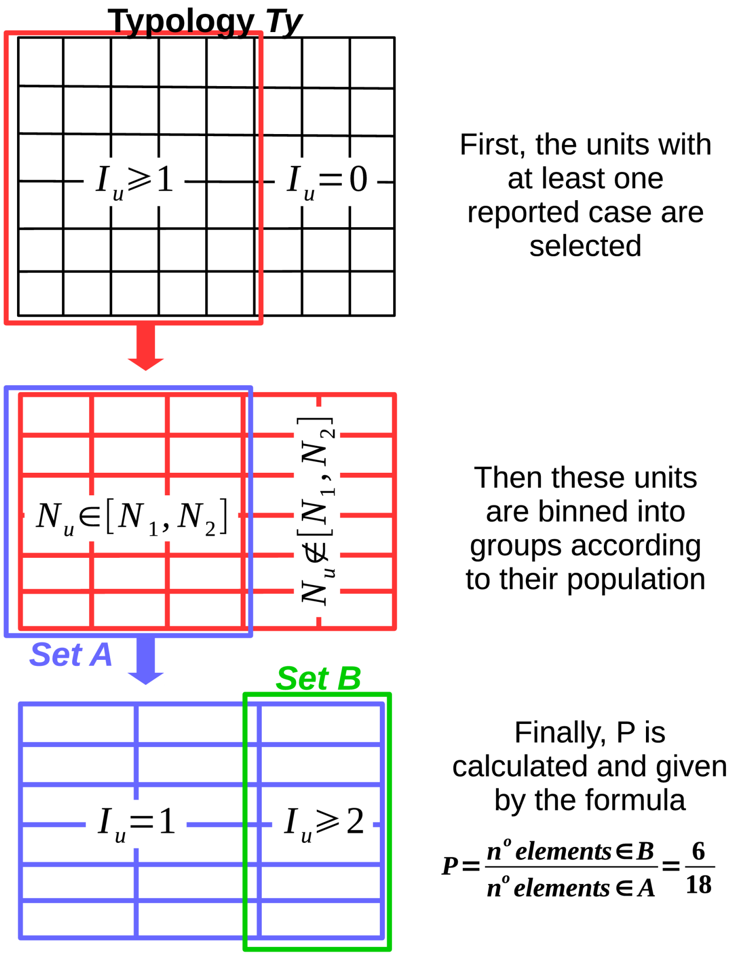

Fig. S1. Diagram illustrating the steps to compute the probability *P* for a given typology.

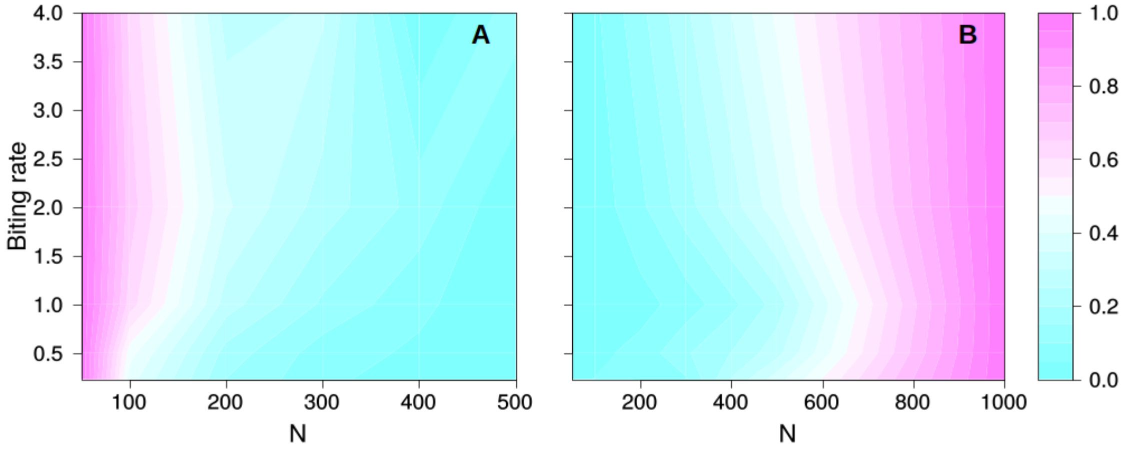

Figure S2. Rescaled force of infection for (A) linear and (B) quadratic *V(N)* for different values of *N* and biting rates. The change of scale is: (*FOI*-min(*FOI*))/max(*FOI*-min(*FOI*)), where minimum and maximum values are with respect to *N* (for a given biting rate). Regardless of biting rate, we observe a decreasing and increasing behavior with *N* for the linear and quadratic cases respectively.

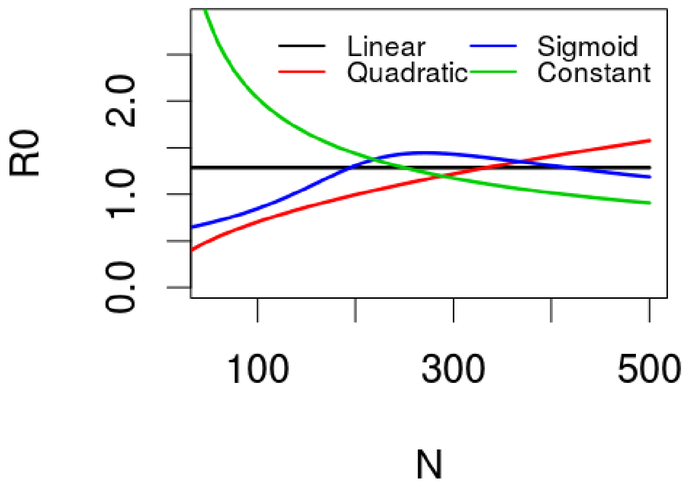

Figure S3. R_0 as a function of *N*.

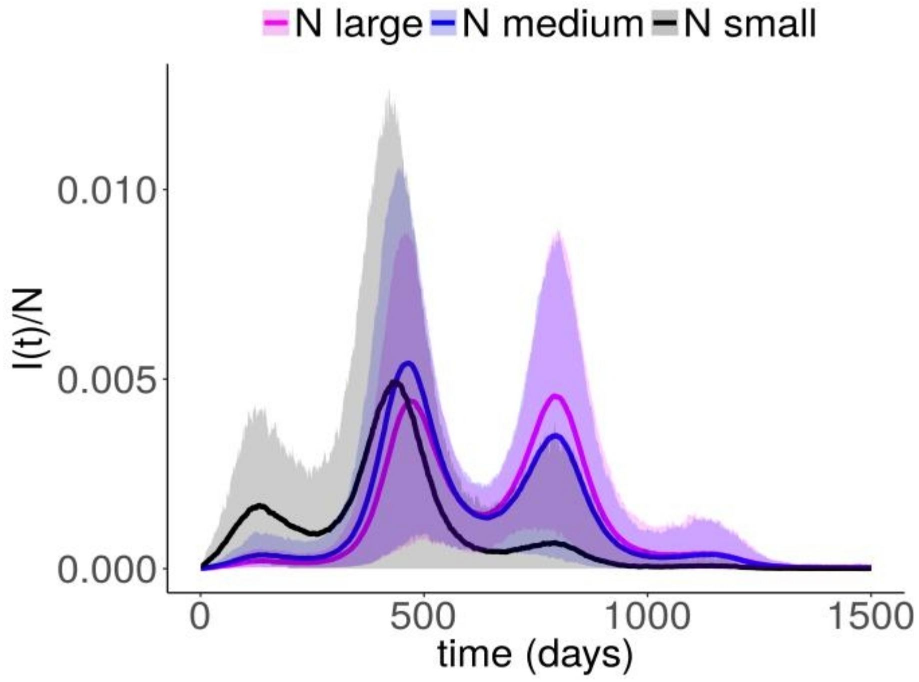

Figure S4. Temporal evolution of incidence for linear *V(N)* in the non-spatial model with temperature forcing (see Fig. 2). Lines correspond to the mean values of incidence and shadow areas, to the 90% confidence intervals. Only simulations with total cumulated cases representing more than 1% of the total host population were included.

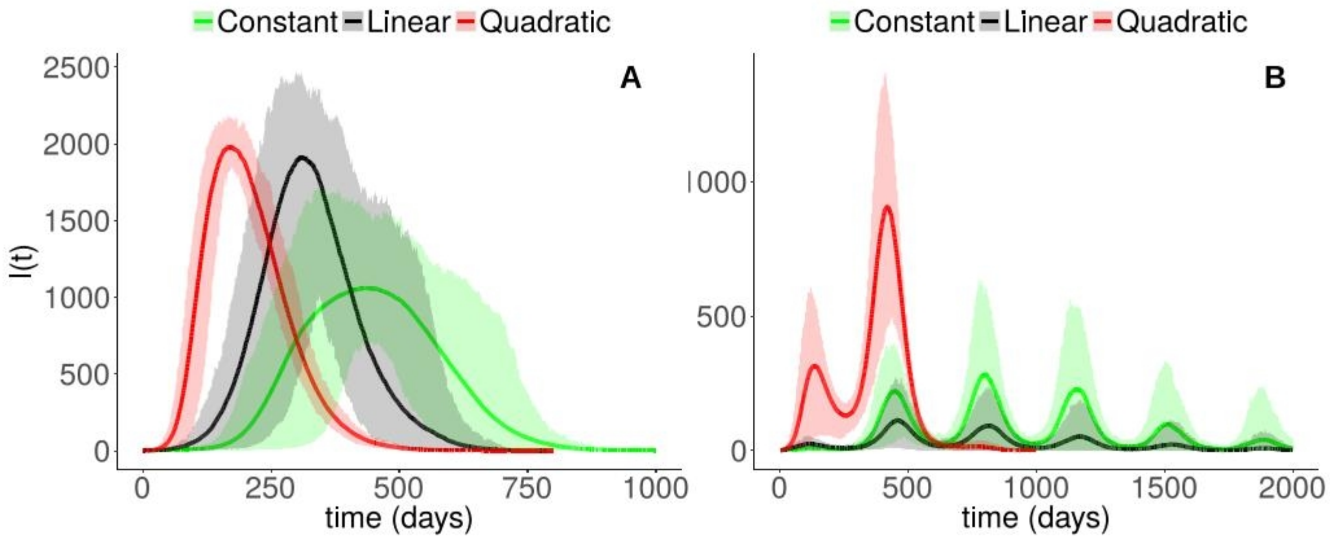

Figure S5. Number of infected hosts as a function of time. In the spatial model, without (A) and with (B) temperature forcing (see Fig. 3 in main text). Lines correspond to the mean values of incidence and shadow areas, to the 90% confidence intervals. Only simulations with total cumulated infected cases representing more than 1% of the total host population were included.

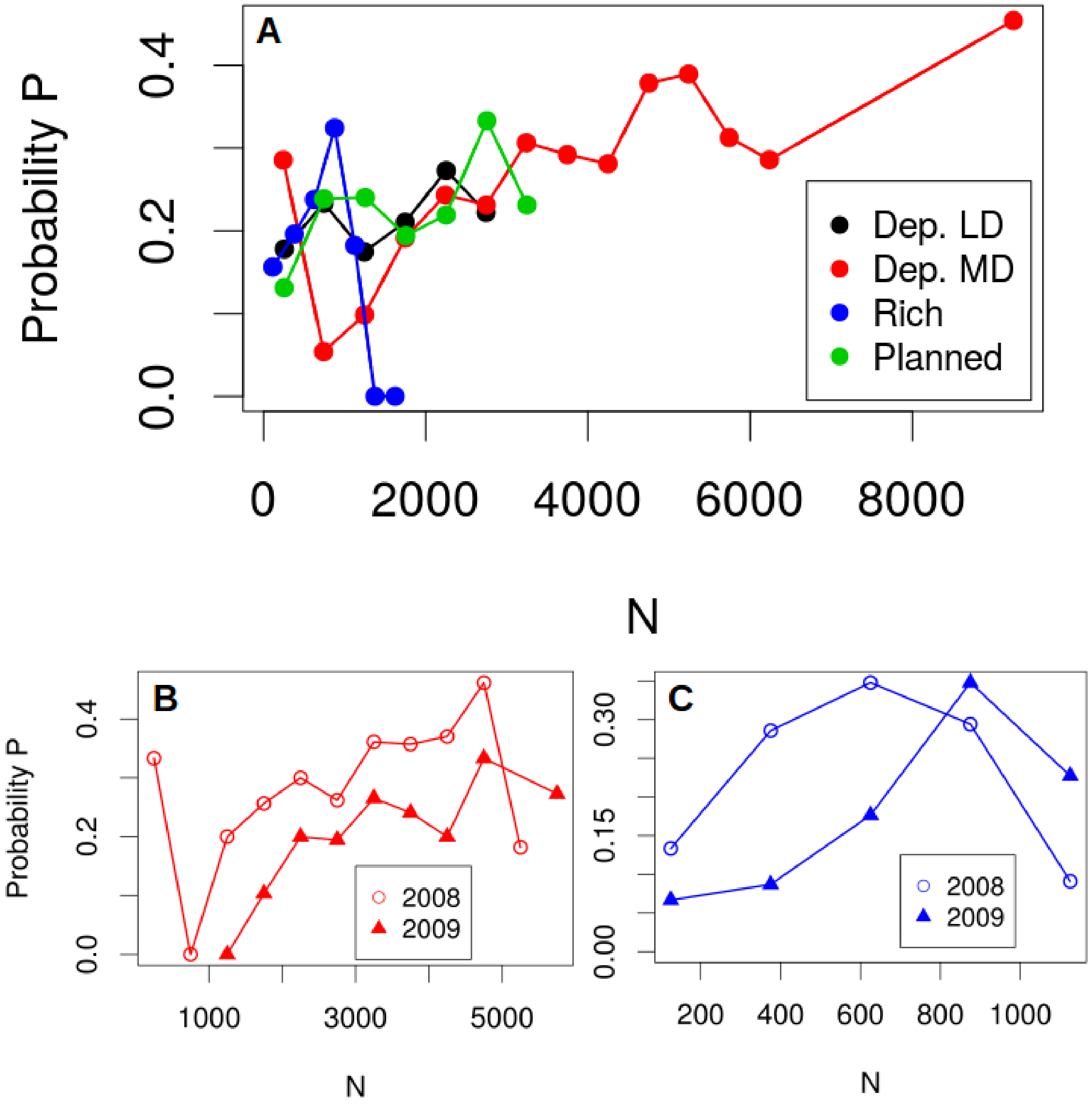

Figure S6. Probability *P* (see main text) as a function of *N* for dengue Delhi data for, Dep. LD (black), Dep. MD (red), Rich (blue) and Planned (green) typologies. A) For years 2008 and 2009 together. Per year for B) Dep. MD and C) Rich typologies. (The Dep. LD data are not shown for the two separate years because the resulting number of points would be too low to address the existence or lack of a trend).

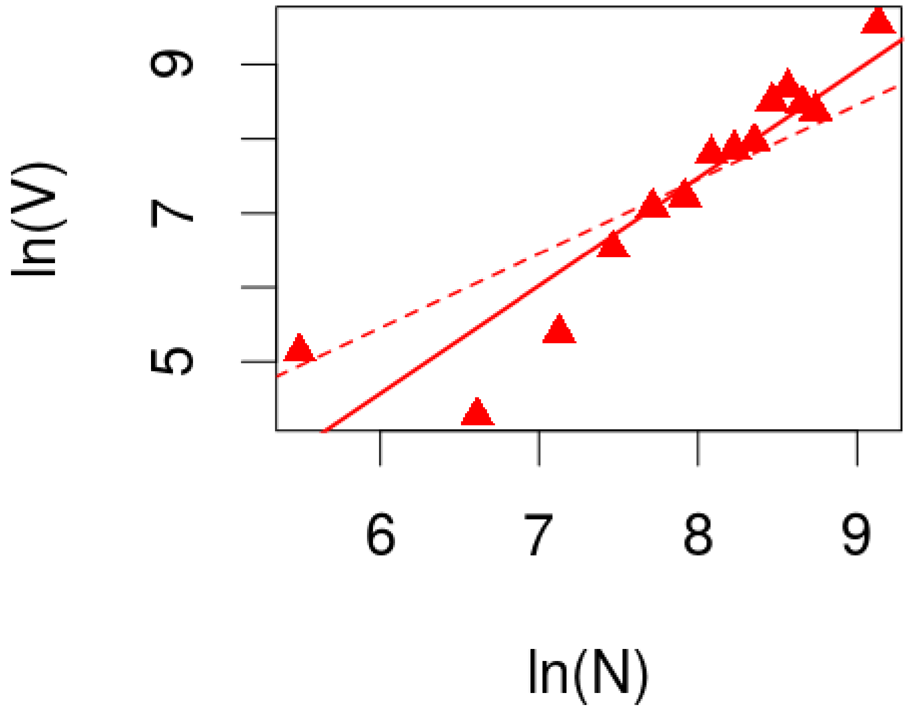

Figure S7. The natural logarithm of *V* is plotted as a function of the natural logarithm of *N* for the Dep. MD typology. The solid line corresponds to the best linear fit and the dashed line to the best fit with a fixed slope of one. In this case, contrary to Fig 4C, the assumed outlier point (first point with the smallest *N*) is taken into account. A slope value of k= 1.45 +/- 0.17 is obtained.
